# C698R mutation in *Lrsam1* gene impairs nerve regeneration in a CMT2P mouse model

**DOI:** 10.1038/s41598-022-15902-3

**Published:** 2022-07-16

**Authors:** Daniel Moiseev, Zafar Wazir, Donghao Liu, Jun Li, Bo Hu

**Affiliations:** 1 Department of Neurology, Wayne State University School of Medicine, Detroit, MI USA; 2 Center for Molecular Medicine and Genetics, Wayne State University School of Medicine, Detroit, MI USA; 3 John D. Dingell VA Medical Center, Detroit, MI USA; 4 Department of Neurology, Houston Methodist Research Institute, Houston, TX USA

**Keywords:** Neuroscience, Genetics of the nervous system, Peripheral nervous system, Regeneration and repair in the nervous system

## Abstract

Missense mutation C694R in the RING domain of the *LRSAM1* gene results in a dominantly inherited polyneuropathy, Charcot-Marie-Tooth disease type 2P (CMT2P). We have generated and characterized a *Lrsam1*^C698R^ knock-in mouse model produced through CRISPR/Cas9 technology. Both heterozygous (*Lrsam1*^+/C698R^) and homozygous (*Lrsam1*^C698/C698R^) knock-in mice exhibited normal motor functions on behavioral tests as well as normal on nerve conduction studies. Axonal density and myelin thickness were not significantly different between mutants and wild-type mice by sciatic nerve morphometric analysis up to 17 months of age. In line with these normal findings, protein–protein interactions between mutant LRSAM1 and RNA-binding proteins (such as FUS and G3BP1) were still present in mouse cells, which differs from the disrupted interactions between these proteins in human CMT2P cells. However, after crush nerve injury, *Lrsam1*^+*/C698R*^ mice had a mild, but statistically significant, reduced compound nerve action potential and conduction velocity during recovery. Therefore, C698R mutation results in a mild impaired nerve regeneration in mice. We speculate that repetitive nerve injuries may, at least partially, contribute to the slowly progressive axonal loss in CMT2P.

## Introduction

Charcot-Marie-Tooth disease (CMT) is a group of inherited peripheral nerve diseases with a collective prevalence of 1:2500, which are caused by monogenic mutations in more than 100 different human genes^1^. Patients with CMT typically present with sensory loss, muscle atrophy, weakness in distal limbs, and foot deformities. Autosomal dominantly inherited CMTs with de-/dysmyelination have been classified as CMT type-1, whereas autosomal dominant CMTs of axonal type are grouped as CMT type-2 (CMT2)^2,3^.

CMT2P is caused by autosomal dominant and recessive mutations in *leucine rich repeat and sterile alpha motif 1* (*LRSAM1*) gene on human chromosome-9^4–12^. Most patients with autosomal dominant CMT2P usually manifest a teenage or adult-onset, slowly progressive, length-dependent sensory-motor axonal polyneuropathy^5,6,11,13^.

LRSAM1 is an E3 ubiquitin ligase with a RING domain at its c-terminal. The RING domain is crucial for the ubiquitination function of LRSAM1. CMT mutations have been shown to disrupt its function^13^. CMT2P was initially found in multiple European families with frame-shift mutations, which altered a large portion of the RING amino acid sequence^7,9,10^. Of the 12 CMT2P-causing mutations, 11 are *dominantly* inherited and located in the catalytic RING domain^13^; which further supports the functional importance of the RING. Our group has described a missense mutation, Cys694Arg, that alters a highly conserved cysteine in the RING and is co-segregated with affected CMT2P members in an American family^6^. This conserved cysteine is required to maintain the structure of E3 ubiquitin ligase through its binding with two zinc ions^4,6,13^. We have further demonstrated that the C694R mutation altered the RNA-binding protein nuclear translocation likely by disrupting the protein–protein interaction between LRSAM1 and the RNA-binding proteins^6^, a potential mechanism in CMT2P pathogenesis.

To further explore this mechanism in vivo, we have generated a *Lrsam1*^C698R^ knock-in mouse model, the amino acid substitution equivalent to the human C69**4**R mutation. Herein, we present our initial characterization of the *Lrsam1*^C698R^ mouse model. We did not observe axonal degeneration in the peripheral nerve in the naïve *Lrsam1*^C698R^ knock-in mice; instead, we found a mild impairment of nerve repair in the mouse model.

## Results

### Generation of ***Lrsam1***^***C698R***^ knock-in mouse

**Figure 1:**
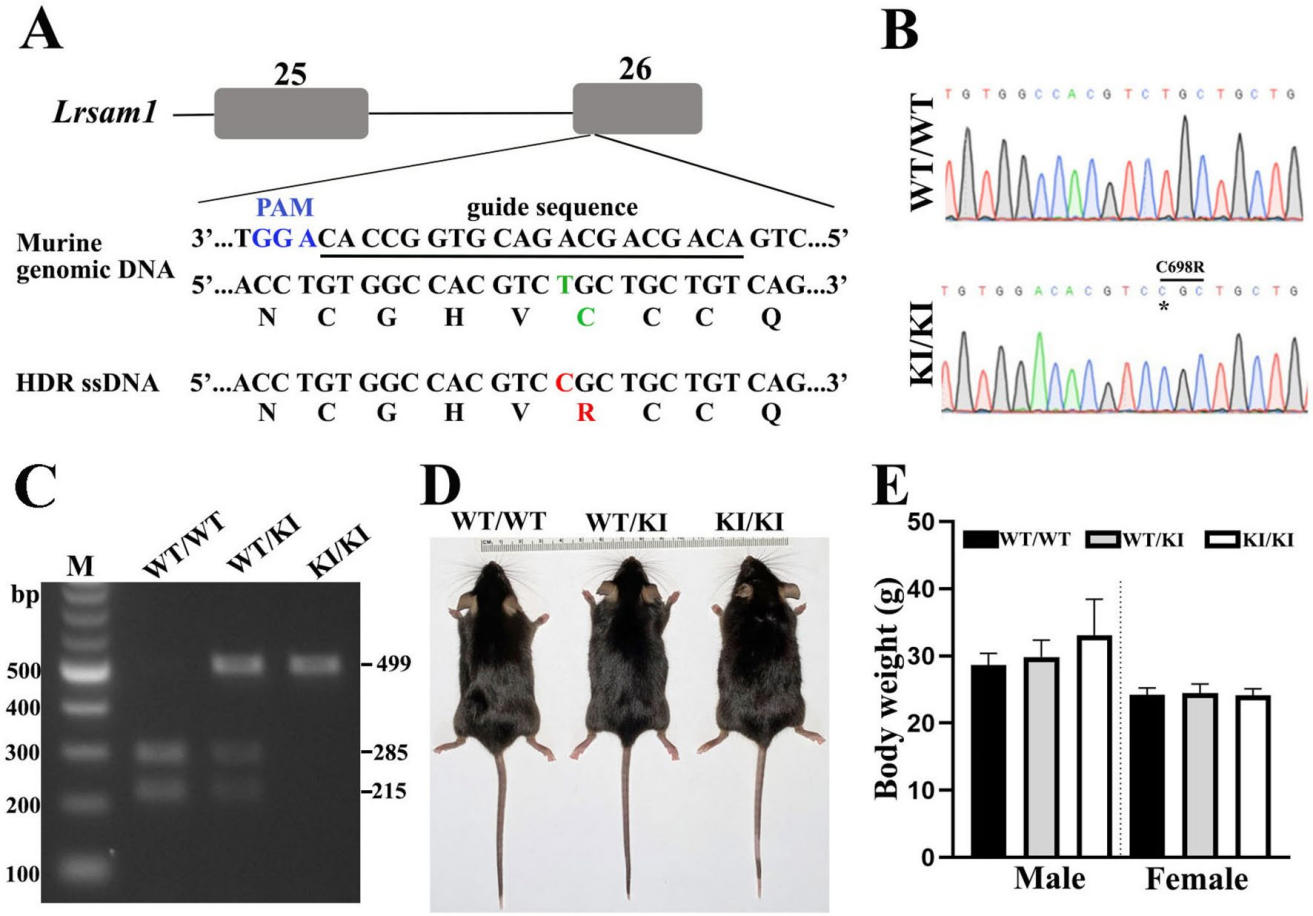
Generation of *Lrsam1*^*C698R*^ knock-in mouse. (**A**) CRISPR/Cas9 (clustered regularly interspaced short palindromic repeats/CRISPR-associated 9) targeting in mouse *Lrsam1* locus. A single guide RNA sequence was underlined. The sequences of amino acid were shown under DNA sequences. The substituted nucleotides were shown in green (wild type) and red (mutation). The PAM (protospacer adjacent motif) sequence was shown in blue. HDR indicates homology-directed repair; ssDNA, single-strand DNA. Exons were represented by gray boxes. (**B**) The *Lrsam1* knock-in allele was identified by sequencing the PCR amplicon surrounding the mutation site. The asterisk indicates the mutation site of knock-in of the *Lrsam1* allele: 5′-TGC(Cys)-3′ > 5′-CGC(Arg)-3′. (**C**) Polymerase chain reaction (PCR) genotyping of *Lrsam1*^*C698R*^ progeny. Mouse DNA segment around the C698 was amplified by the following primers and then digested with a restriction enzyme Mlsl recognizing tgg/cca. The PCR products were two bands at 285 bp and 215 bp for WT, one band at 499 bp for *Lrsam1*^*C698R*^. Lane 1 = DNA marker (M); Lane 2 = *Lrsam1*^+*/*+^ (WT/WT); Lane 3 = *Lrsam1*^+*/C698R*^ (KI/WT); Lane 4 = *Lrsam1*^*C698R/C698R*^ (KI/KI). (**D**) External physical analysis. 6-month-old WT/WT, KI/WT, and KI/KI littermates were placed next to a centimeter ruler. (**E**) At 14–16 months of age, we observed no significant difference in body weight between wild-type and mutant mice (*P* > 0.05, n = 5 in each group).

The missense mutation c.2080T > C (C694R) in human *LRSAM1* that we described^6^ was introduced into the mouse genome through CRISPR/Cas9-mediated mutagenesis (Fig. 1A). Compared with the human sequence surrounding the c.2080T position, we found a C69**8**R substitution in the mouse *Lrsam1* gene equivalent to the human C69**4**R mutation (Fig. 1A). Heterozygous (*Lrsam1*^+*/C698R*^) mice were crossed to obtain homozygous (*Lrsam1*^*C698R/C698R*^) mice. DNA sequencing revealed that the mutant mice carry both C698R and a silent C to A mutation 7 bp upstream to C698R mutation (Fig. 1B). Mutant mice were further verified by restriction fragment length polymorphism analysis to determine whether *Lrsam1* knock-in occurs in both alleles or one allele (Fig. 1C). These knock-in mice did not appear to differ from *Lrsam1*^+*/*+^ mice in appearance and body weight (Fig. 1D,E).

### Behavioral studies showed no abnormality in *Lrsam1*^*C698R*^ mice

**Figure 2:**
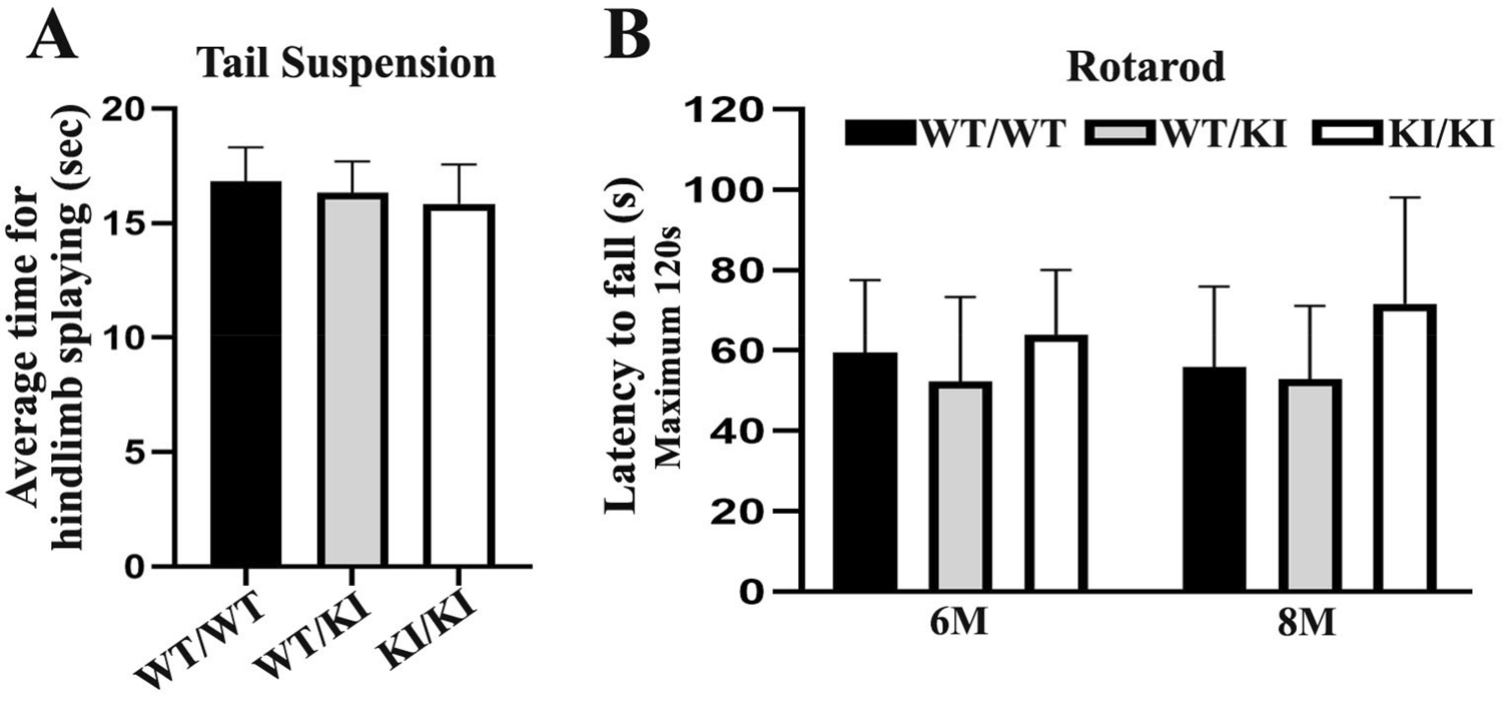
Behavioral analysis of *Lrsam1*^*C698R*^ knockin mice. (**A**) Quantification of hindlimb clasping test at 12 months old. Animals were held at tails for 20 s, and time displaying hindlimb splaying was recorded. Data presented as the average time for hindlimb splaying. Average time on the hindlimb splaying was not different in *Lrsam1*^+*/C698R*^ and *Lrsam1*^*C698R/C698R*^ mice, compared to their *Lrsam1*^+*/*+^ littermates (*P* > 0.05, n = 6 for each genotyping group). (**B**) Rotarod performance showed the average time, in seconds (s), spent on the accelerating rotating rod of 6 and 8 months of age in *Lrsam1*^+*/C698R*^, *Lrsam1*^*C698R/C698R*^*,* and their *Lrsam1*^+*/*+^ littermates. The mutant mice showed no significant decline in the locomotor performance (*P* > 0.05, n = 6–9 for each genotyping group).

Rotarod and hindlimb clasping test assess mouse motor coordination and limb weakness. At 12 months of age, *Lrsam1*^+*/C698R*^* and Lrsam1*^*C698R/C698R*^ mice did not show any abnormalities in hindlimb clasping or paw extension during tail suspension (Fig. 2A) when compared with *Lrsam1*^+*/*+^ mice. The fall latency from the rotating bas of Rotarod was not different among *Lrsam1*^+*/*+^, *Lrsam1*^+*/C698R*^* and Lrsam1*^*C698R/C698R*^ mice measured at 6- and 8-months of age (Fig. 2B).

### The nerve conduction study was not significantly different between mutants and wild-type mice

**Figure 3:**
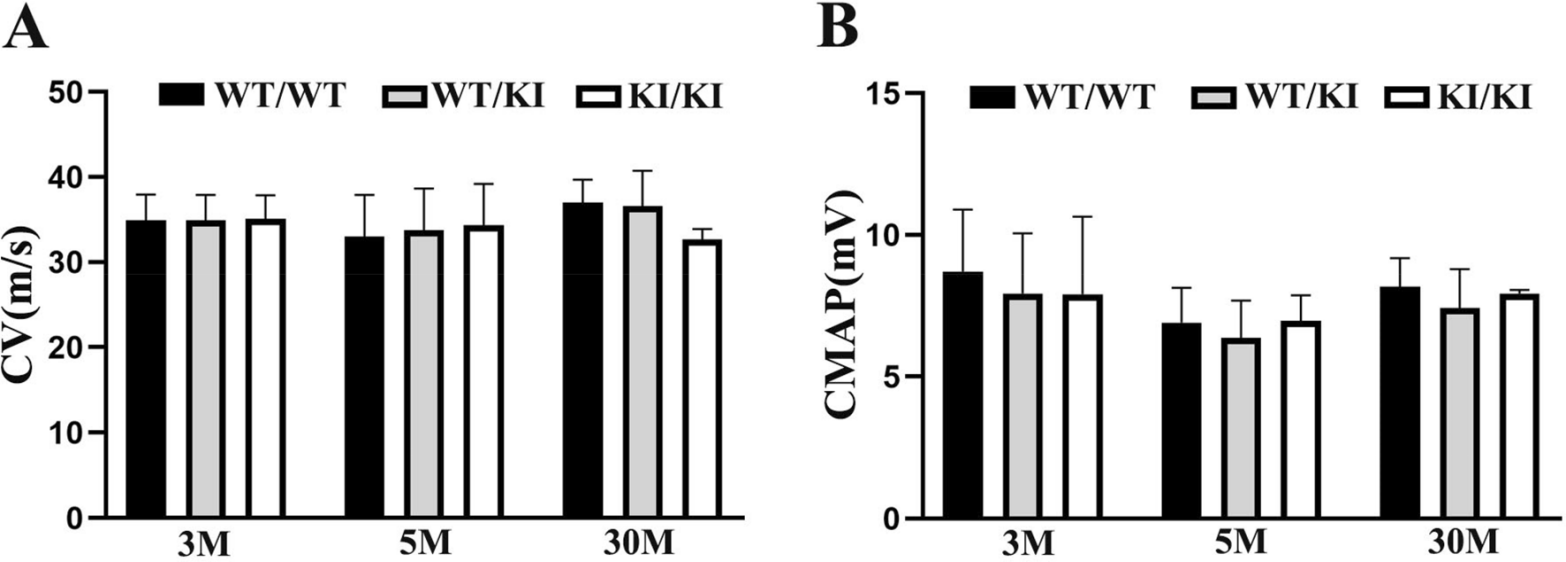
Nerve conduction studies (NCS) at 3, 5, 30-month-old WT and mutant mice. (**A**) NCS showed no significant difference in conduction velocity (CV) between *Lrsam1*^+*/*+^ (WT) and *Lrsam1*^+*/C698R*^ (KI/WT) or *Lrsam1*^*C698R/C698R*^ (KI/KI) mice tested in separate cohorts at 3, 6 and 30 months of age (*P* > 0.05, n = 18, 30, 13 for 3-month-old, 8, 11, 6 for 5-month-old, 7, 9, 3 for 30-month-old, *Lrsam1*^+*/*+^*, Lrsam1*^+*/C698R*^*, Lrsam1*^*C698R/C698R*^ mice). (**B**) Compound muscle action potential (CMAP) was also normal in mutants.

We measured compound muscle action potentials (CMAP) at the paw muscles and conduction velocities (CV) on the sciatic nerves. CMAPs and CVs were not significantly different between *Lrsam1*^+*/*+^, *Lrsam1*^+*/C698R*^ and *Lrsam1*^*C698R/C698R*^ mice up to 2.5 years of age (*P* > 0.05, Fig. 3A,B).

### Myelin and axon were morphologically normal in *Lrsam1*^*C698R*^ mice

**Figure 4:**
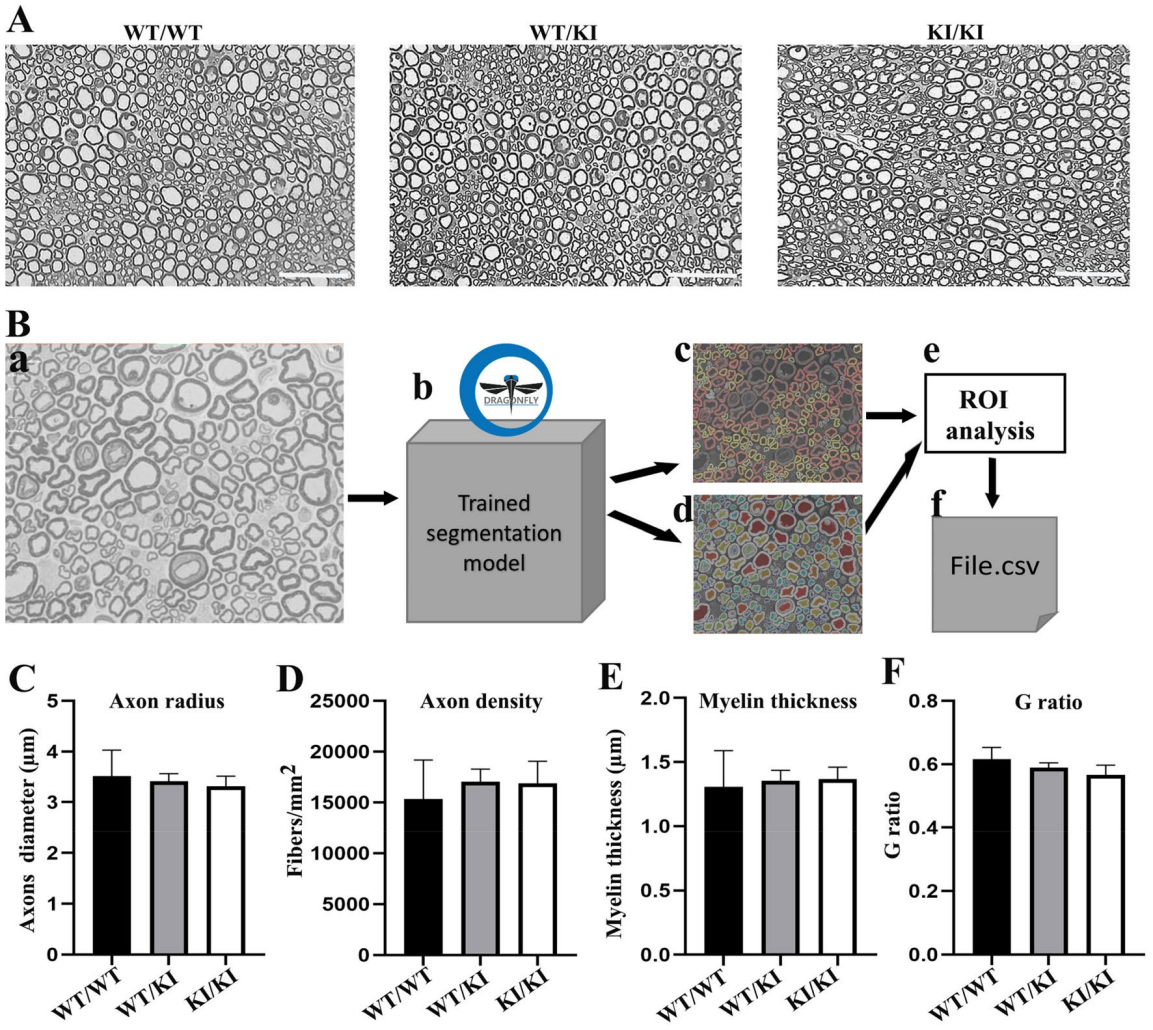
Morphometric analysis for *Lrsam1*^*C698R*^ mouse sciatic nerve. (**A**) Semithin sections of sciatic nerves showed myelinated nerve fibers were morphologically similar between 17-month-old wild-type and *Lrsam1*^+*/C698R*^ or *Lrsam1*^*C698R/C698R*^ mice. Scale bars = 50 µm. (**B**) 40 × Bright Field Image (a) is processed by an ORS Dragonfly trained model (b), which produces a segmentation of myelin (c) and inner axonal areas (d). The software’s Region of Interest analysis tool is used to measure each fiber’s inner and outer areas (e). The results are exported into a spreadsheet, where myelin radii, thickness, and g-ratio are calculated (f). (**C–F**) Morphometric analysis showed that the axon radius, axon density, myelin thickness, and g-ratio (determined by dividing axonal radius by the outer fiber diameter) were not significantly different between 17-month-old wild-type and *Lrsam1*^+*/C698R*^ or *Lrsam1*^*C698R/C698R*^ sciatic nerves (*P* > 0.05, t-test, n = 5 per group, 3 males and 2 females each genotype).

We further studied mouse nerves morphologically. Axons and myelin were examined and quantified at 17 months of age in the sciatic nerves. Visual inspection on semithin sections did not find axonal degeneration or demyelination features, such as loss of axons, myelin debris, and clusters of regenerating axons (Fig. 4A). Myelinated nerve fibers on the cross-sections of the sciatic nerves were quantified using deep-learning-based analysis^14^ (Fig. 4B). There was no significant difference in axon diameter, fiber density, g-ratio, and myelin thickness in 17-month-old *Lrsam1*^+*/C698R*^ and *Lrsam1*^*C698R/C698R*^ sciatic nerves, compared with those in wild-type mice (Fig. 4C,F).

### Protein–protein interaction between LRSAM1 and RNA-binding proteins was not disrupted by the C698R mutation

**Figure 5:**
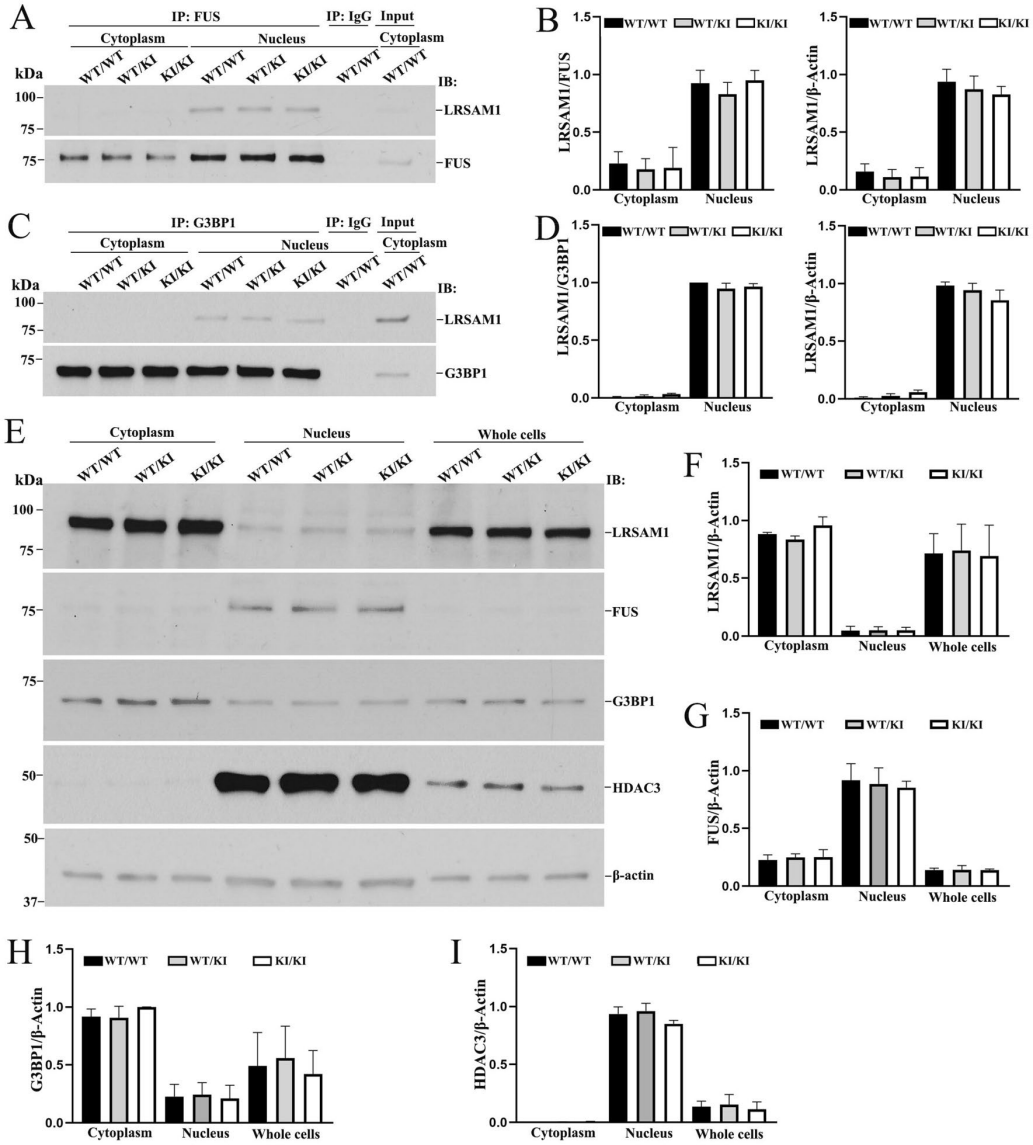
C698R *Lrsam1* mutation fails to disrupt interactions between LRSAM1 and RNA-binding proteins. (**A**) Cytoplasmic and nuclear protein fractions were extracted from *Lrsam1*^+*/*+^, *Lrsam1*^+*/C698R*^ and *Lrsam1*^*C698R/C698R*^ fibroblasts. Proteins were immunoprecipitated (IP) with anti-FUS antibody, and the precipitated proteins were immunobloted (IB) with anti-LRSAM1, anti-FUS antibody (IP lines). IgG was used as a IP negative control. Cell lysate was loaded as inputs. FUS antibody was able to pull down LRSAM1 in nuclear fractions of wild-type and C698R mutation fibroblasts. (**B**) Intensities of protein bands in co-IP experiments were measured using ImageJ (n = 3). Anti-LRSAM1 signals were normalized by either anti-FUS signals or anti-β-Actin signal. LRSAM1 levels displayed no significantly difference between wild-type and mutant cells (*P* > 0.05). Thus, C698R failed to disrupt the protein-protein interactions between LRSAM1 and FUS. (**C**) Cytoplasmic and nuclear protein fractions were separately used for IP with an anti-G3BP1 antibody and then blot with an anti-LRSAM1 and an anti-G3BP1 antibody. G3BP1 antibody was able to pull down LRSAM1 in nuclear fractions of both wild-type and C698R fibroblasts. (**D**) Intensities of LRSAM1 and G3BP1 protein bands were quantified using ImageJ. LRSAM1 levels were not significantly different between wild-type and mutant cells (*P* > 0.05). Thus, C698R mutation did not disrupt LRSAM1 interaction with G3BP1. (**E**) Cytoplasmic, nuclear proteins and whole cell were extracted from the mouse fibroblasts and examined by Western blot for levels of LRSAM1, FUS and G3BP1. HDAC3 served as a loading control of the nuclear extracts. β-actin was included as an overall loading control. (**F–I**) Histograms show the signal intensity of protein bands in arbitrary units after their normalization with the level of β-Actin. Each bar represents the mean of triplicate values. The C698R mutation did not significantly decrease the level of LRSAM1, FUS, G3BP1, or HDAC3 protein when compared with those in wild-type cells (*P* > 0.05).

Our previous study in fibroblasts from patients with CMT2P showed that C694R mutation disrupts the protein–protein interactions between LRSAM1 and RNA-binding proteins (including FUS and G3BP1), leading to altered nuclear-cytoplasmic translocation of the RNA-binding proteins, a potential mechanism of neuronal degeneration in CMT2P^6^. To determine whether similar molecular abnormalities occur in mouse fibroblasts, we performed co-IP. We found that interactions were still present and not significantly different between FUS-mutant LRSAM1 and FUS-wild type LRSAM1 or between G3BP1-mutant LRSAM1 and G3BP1-wild type LRSAM1 (*P* > 0.05, Fig. 5A–D). There were no significant differences in nuclear / cytoplasmic ratios among LRSAM1, FUS and G3BP1 protein levels (*P* > 0.05, Fig. 5E–I). This finding differs from disrupted interactions between mutant LRSAM1 and RNA-binding proteins in human cells from patients with CMT2P.

### Nerve regeneration was mildly impaired in *Lrsam1*^*C698R*^ nerves

Most patients with CMT2P are due to a heterozygous *C694R* mutation^6^*.* We thus examined nerve regeneration using crush-nerve injuries on *Lrsam1*^+*/C698R*^ and *Lrsam1*^+*/*+^ mice that were age- and sex-matched. A baseline NCS found no difference in CMAP or CV between the two genotypic groups. After the nerve injury, NCS was done every 2–3 weeks. Sciatic nerves were dissected at 23 weeks after the second surgery or 43 weeks after the initial surgery respectively for morphometric analysis.

**Figure 6:**
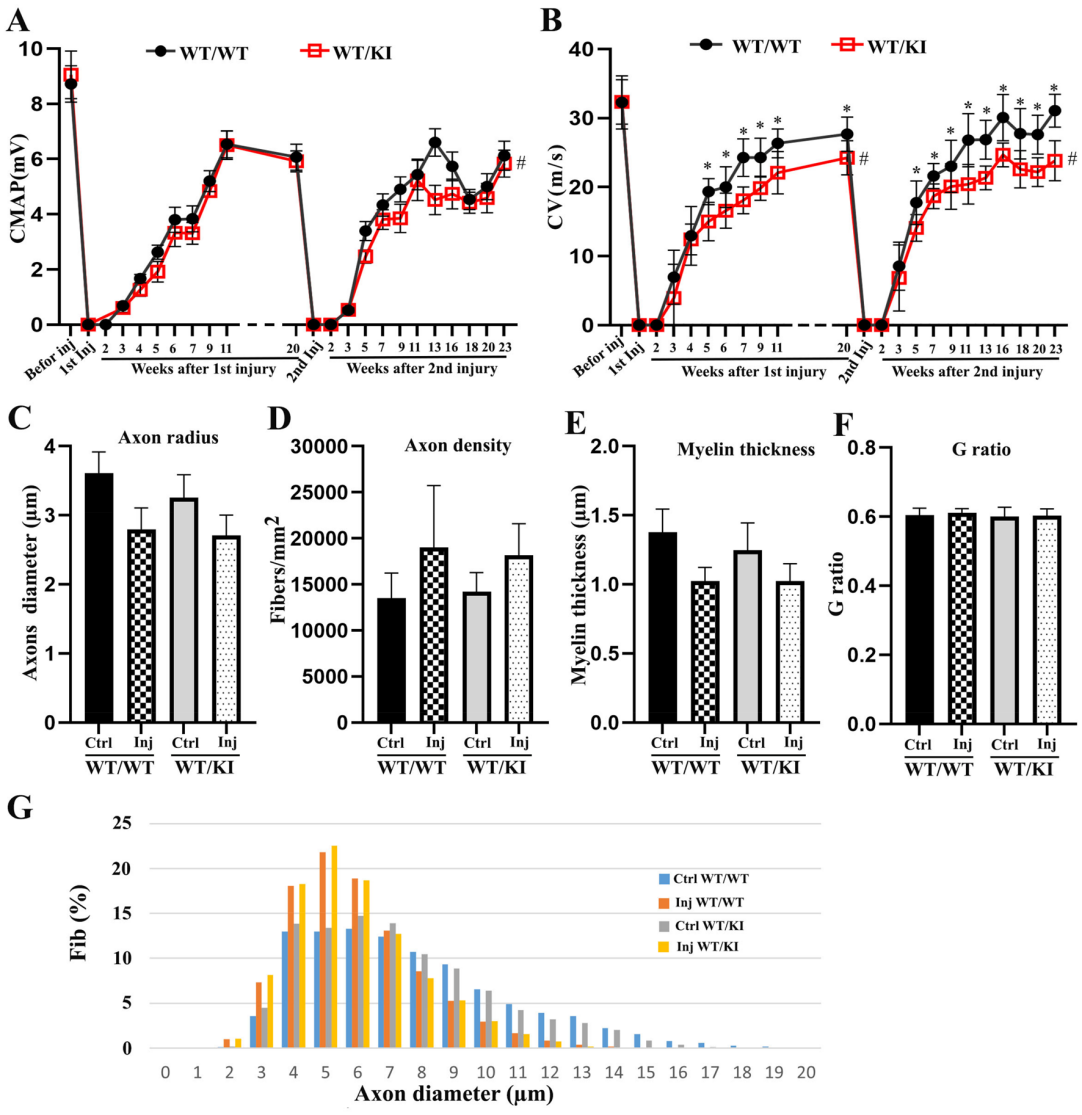
Nerve regeneration was slightly, but statistically significant, impaired in the *Lrsam1*^*C698R*^* mice*. (**A**) NCS was carried out before the surgery, 2 weeks post-surgery, and every 1–2 weeks afterward until 11 weeks. At 20 weeks, another baseline NCS was measured before the 2nd injury was done. NCS was then conducted every 2–3 weeks, until sciatic nerves were collected for morphometric analyses at 23 weeks, or 43 weeks after the initial surgery respectively. Baseline measurements showed no difference in CMAP between the genotypes (n = 15 for *Lrsam1*^+*/*+^ group, 12 for *Lrsam1*^+*/C698R*^ group, *P* = 0.763). The linear mixed model showed a significant negative effect of *Lrsam1*^+*/C698R*^ genotype on CMAP: − 0.516 mV over the whole period excluding the baseline, (*P* < 0.001), − 0.417 mV over the first postoperative period (*P* = 0.07), and − 0.606 mV over the second post-op period (*P* < 0.001). Time overall had a positive effect on CMAP, associated with 0.192 mV increase per time point in all animals (p < 0.001), 0.715 mV during the 1st post-op period (*P* < 0.001), 0.501 mV during the 2nd post-op period (*P* < 0.001). Interactions of time and genotype were not found to be significant. # = *P* < 0.001. (**B**) CV baseline measurements showed no significant difference between the genotypes n = 15 for *Lrsam1*^+*/*+^ group, 12 for *Lrsam1*^+*/C698R*^ group, *P* = 0.961). A linear mixed model showed a significant negative effect of *Lrsam1*^+*/C698R*^ genotype on CV: − 7.24 m/s over the whole time excluding the baseline, (*P* < 0.001), − 3.41 m/s over the first postoperative period (*P* < 0.001), and − 7.25 m/s over the second post-op period (*P* < 0.001). Time overall had a positive effect on CV, associated with 0.55 m/s increase per time point overall in all animals (*P* < 0.001), 1.868 m/s during the 1st post-op period (*P* < 0.001), 1.638 m/s during the 2nd post-op period (*P* < 0.001). Interactions of Genotype x Time were found to have a significant effect on CV and indicated a slowed rate of CV recovery in *Lrsam1*^+*/C698R*^ mice during the whole time period (*Lrsam1*^+*/*+^ increased 0.641 mV (*P* < 0.001), *Lrsam1*^+*/C698R*^ increased 0.437 mV (*P* < 0.001), interaction *P* < 0.001), over the first postoperative period (*Lrsam1*^+*/*+^ increase 2.078 mV (*P* < 0.001), *Lrsam1*^+*/C698R*^ increased 1.628 mV (*P* < 0.001), interaction *P* < 0.001), and over the second post-op period (*Lrsam1*^+*/*+^ increased 1.906 mV (*P* < 0.001), *Lrsam1*^+*/C698R*^ increased 1.294 mV (*P* < 0.001), interaction *P* < 0.001). # = p < 0.001. CV was significantly different between 5- and 20-weeks post-surgery. * = *P* < 0.001. (**C–E**) Sciatic nerves were collected from the surgery and control hindlimbs, embedded in Epon resin, sliced and imaged under a brightfield microscope at 40X magnification. Automatic segmentation of myelin was performed and refined in Dragonfly software. Results were analyzed using a mixed linear model and showed that crash nerve surgery was associated with an increase of axon density (+ 4758.8 fibers/mm^2^), a decrease of axon radius (− 0.74 µm), and a decrease of myelin thickness (− 0.30 µm), all statistically significant (*P* < 0.05). Genotype or interaction between genotype and surgery status were not found to be statistically significant. (**F–G**) Morphometric analysis of the nerve fibers revealed no significant difference in g-ratio and axon-diameter distribution in injured *Lrsam1*^+*/C698R*^ nerves as compared to injured *Lrsam1*^+/+^ nerves (n = 6 per group, 4 males and 2 females each genotype). Ctrl = Control. Inj = crush injury.

*Lrsam1*^+*/C698R*^ mice had a statistically significant negative main effect on CMAP over the entire period (-0.516 mV), and during the second recovery period (− 0.606 mv) (Fig. 6A). The main effect of mutant on CV was statistically significant during the post-surgery periods: − 7.24 m/s over the whole time period, − 3.41 m/s during the first recovery, and − 7.25 m/s during the second recovery (Fig. 6B). These findings suggest an incomplete recovery of CMAP and CV after the injury^15^.

Time had a statistically significant positive effect on CMAP and CV in both genotypes, indicating an effect of recovery process. More importantly, CV recovery showed a statistically significant genotype x time interaction over the two recovery periods, with *Lrsam1*^+*/*+^ recovering faster than *Lrsam1*^+*/C698R*^ mice (Fig. 6A,B). In addition, *Lrsam1*^+*/C698R*^ also showed a reduced rate of CV recovery as compared to *Lrsam1*^+*/*+^ mice.

Morphometric analysis showed that surgeries had main effects on axon density (an increase of 4758.8 fibers/mm^2^), a decrease of axon radius (− 0.74 µm), and a decrease of myelin thickness (− 0.30 µm), all statistically significant changes expected in regenerating nerves (*P* < 0.05). However, comparisons between *Lrsam1*^+*/*+^ and *Lrsam1*^+*/C698R*^ nerve morphometrics, as well as the interaction of genotype and injury status were not significantly different (Fig. 6C–G).

Additionally, in homozygous C698R mice, CV and CMAP were not significantly different from that of heterozygous C698R mice after injury; but were significantly lower than that of wild-type mice (Supplementary Fig. 1A–B). Again, the morphometric analysis did not find a significant change in homozygous injured nerves, compared with heterozygous and wild-type injured nerves (Supplementary Fig. 1C–F).

Taken together, C698R *Lrsam1* mutation only mildly impaired nerve function during regeneration.

## Discussion

In this study, we have shown the production and initial characterization toward a new mouse model of CMT2P, where a knock-in of the C698R *Lrsam1* mutation was introduced into the mouse genome. However, we observed no peripheral neuropathy in naïve *Lrsam1*^+*/C698R*^ and *Lrsam1*^*C698R/C698R*^ mice up to 2.5 years of age but did find a mildly impaired nerve function during regeneration after nerve injury. While the electrophysiological phenotype is subtle, it could contribute to the slowly progressive polyneuropathy in patients with CMT2P. As every person is exposed to many nerve injuries during their lifetime, we speculate that an accumulation of impaired nerve recoveries may eventually contribute to the polyneuropathy of CMT2P^16–18^.

This finding also brings up a challenging issue. The nerve abnormality in the knock-in mice was disproportionally subtle, comparing with the polyneuropathy in persons with CMT2P^6^. This mild phenotype is in line with absent disruption of protein–protein interactions between mutant LRSAM1 and RNA-binding proteins (Fig. 5). The exact reason for the loss of this disruption of protein–protein interaction by the mutation in mice is unclear at this point. However, there have been many precedents where mouse models could not completely recapitulate phenotypes in its human counterpart^19^. Perhaps, CMT2P needs to be modeled in larger animals.

Notice that we did not find an age-effect in the NCS of 30-month-old mice, while CV is known to decline over time^20^. This is not unexpected. Age-effect in CV is typically detectable in consecutive recordings in same animals. This was not possible in this study as tissues had to be collected at different ages; thus, our CV data in the 30-month-old mice were cross-sectional; thus may and may not show the expected age-effect. However, it would not affect the overall conclusion of this study. This was not likely a technical issue as the same NCS technique was used in our studies before and has detected age-effect^21^.

Another potential limitation relates to small n-numbers of some experimental groups. While our data do not provide evidence of obvious progressive neuropathy in mutant mice without the challenge of nerve injury**,** subtle alterations could be undetected due to the small n-numbers. However, impaired nerve regeneration after injury was reproduced in repeated experiments and also in a different experimental group (Fig S1), supporting the key conclusion of this study.

In summary, we have produced a knock-in mouse with C698R mutation in order to model CMT2P. While the phenotype is not robust, mild abnormality in nerve repair provides a helpful clue toward the slowly progressing polyneuropathy in CMT2P.

## Methods

### Animal generation

The *Lrsam1*^*C698R*^ knock-in mice were generated on a C57Bl/6J background using the CRISPR/Cas9 technique. Briefly, single-cell zygotes from C57BL/6J mice were microinjected with mRNA encoding Cas9 and a guide sequence (5′-ACAGCAGCAGACGTGGCCAC-3′ at 20 ng/µl) to target the exon 26 of *Lrsam1*. A single stranded DNA oligo carrying the C698R mutation was co-injected at 20 ng/µl to promote homology-directed repair. The sequence of the oligo was (…ACGTCCGCTG…). The underlined "C" changes the first base of codon 698, creating the C to R substitution. Following implantation of the embryos into surrogate females, the microinjection resulted in 49 live-born mice. Fourteen of them carried idles consistent with non-homologous end joining (NHEJ) events. Two female offspring carried the C698R, as well as an upstream silent mutation. These mice were bred with wild-type C57BL/6 J males to establish the germline transmission of the mutation. The offspring were confirmed to carry both the C698R and the silent C to A mutation 7 bp upstream to C698R mutation. The silent mutation was likely a not-targeted change accidentally introduced by the genetic manipulation as we did not find this silent mutation in the progeny of *Lrsam1*^+/+^ mice. After germline transmission of the targeted variant allele, we used the congenic *Lrsam1*^+/C698R^ mice derived from two founders to backcross with C57BL/6J mouse for at least 10 generations. A mixed mouse line from both founders were used for experiments. The experimental mice were obtained by intercrossing heterozygous mice (*Lrsam1*^+/C698R^) and were confirmed by both restriction fragment length polymorphism analysis and sequencing. The mice used in this study were age-matched and sex-mixed littermates. Mice were maintained on a standard 12 h light/12 h dark cycle, ad libitum access to food and water, and housed in plastic cages with standard rodent bedding. We did not observe any difference in mortality between genotypes.

### Animal ethics statements

All animal experiments and procedures were reviewed and approved by the Institutional Animal Care and Use Committee (IACUC) at the Wayne State University and were performed in accordance with federal and university guidelines and regulations for the care and use of experimental vertebrate animals. Authors have complied with the ARRIVE guidelines for reporting.

### Mouse PCR genotyping

DNA was extracted from ear clips by incubation in 100 μl DirectPCR Lysis buffer (Cat # 102T, Viagen) with 2 μl of 10 mg/ml proteinase K (Cat #P4850, Sigma) at 55 °C overnight. After deactivating the mixture at 85 °C for 45 min, the supernatant was used for genotyping by PCR. DNA segments around the C698 were amplified by the following primers: forward primer, 5′-CCA GGT AAG CAG TAC ACG CCT G-3′ and reverse primer 5′-GGT GAC AAA GGC CTA TGG CAG T-3′. PCR was programmed as follows: 94 °C, 5 min; (94 °C, 30 s; 60 °C, 1 min; 72 °C, 1 min) × 30 cycles; 72 °C, 6 min; 4 °C, 2 min. PCR products were digested with a restriction enzyme Mlsl recognizing tgg/cca at 37 °C for 3 h, deactivated at 65 °C for 20 min, and then fragments were imaged on an agarose gel. The wild-type (WT) allele produced two bands at 285 bp and 215 bp. Due to the silent mutation, the mutant allele only gave a band at 499 bp.

### Rotarod test

Wild-type *Lrsam1*^+*/*+^ and *Lrsam1*^*C698R*^ knock-in mice were placed on an accelerating (0.1 rpm/second) rotarod (Columbus Instruments, Columbus, OH) that progressed from 16 to 28 rpm over 2 min. If mice jumped off, turned around, or hung to the rod, they were given an additional attempt for the trial (up to a maximum of 3 attempts for any given trial). Once mice were in position, facing the correct direction, the device was turned on, and they walked until they lost balance and fell off or until the 2-min run-time ended. All mice were given 2 days of training prior to the test. Each mouse underwent 3 trials a day for 3 days following the training, with each trial separated by 30-min rest. The results were averaged for each animal. The cohorts of mice at 6- and 8-months of age were two separate groups; and were tested concurrently on the Rotarod. This was not a longitudinal test on the same group of mice at two different time points.

### Hindlimb clasping test

Mice were held by the tail 30 cm above a tabletop, and video-recorded for 20 s. Abnormal postures such as hindlimb clasping and sustained straining of paws were recorded. Videos were analyzed by a researcher blinded to the genotype. The time span for clasping and paw straining was also calculated.

### Nerve conduction studies (NCS)

NCS data were acquired as described^21^. Mice were anesthetized with isoflurane. CMAP was recorded from the intrinsic foot muscles using needle electrodes. Stimulation electrodes were placed in the mouse sciatic notch and at the ankle and recording was made on the paw. Supramaximal stimulations at each stimulation point evoked CMAP; and conduction velocity was calculated between the two stimulation points.

### Sciatic nerve semithin section

This technique has been described previously^22^. Sciatic nerves were dissected and fixed in 4% paraformaldehyde and 2.5% glutaraldehyde mixture. Nerves embedded in Epon were sectioned to 1 µm thickness. The entire field of transverse sections of each nerve was imaged for morphometric analysis.

### Nerve morphometrics

40X light images of mouse sciatic nerves were taken using a Leica THUNDER microscope. The segmentation model was developed based on a U-net architecture^14^. U-net architecture consists of interconnected expanding and contracting paths. This allows the model to localize image features, while retaining the original image detail. The model was implemented in ORS Dragonfly software, trained with ~ 150 mouse sciatic nerve images and their respective myelin segmentations. Extensive data augmentations of training were applied to the images, including image scaling, rotation, and other transformations. This approach allowed for more robust learning. The trained model was applied to the images to generate segmentations with isolated myelin and axons from the background. Each axon's inner and outer areas were measured. Then, each fiber’s diameter, myelin thickness, and g-ratio were calculated.

### Primary mouse fibroblast culture

The primary fibroblasts were derived from toe skins of 2-month-old *Lrsam1*^+*/*+^,*Lrsam1*^+*/C698R*^ and *Lrsam1*^*C698R/C698R*^ mice. The tissue homogenate was incubated in DMEM high-glucose medium (Cat# 11995, Thermo Fisher Scientific) containing 20% FBS and 0.6 mg/ml collagenase II (Cat# LS004205, Worthington Biochemical) for 12 h at 37 °C incubator. Thereafter, cells were pelleted by centrifugation, washed with PBS and cultured in DMEM/10% FBS at 37 °C in a humidified atmosphere containing 5% CO_2_. Fibroblast outgrowth started at day 3–5. For the experiments, we used cells out of the first three passages of the primary culture.

### Co-immunoprecipitation (co-IP) and immunoblotting

This technique has been described^6^. Whole-cell proteins were extracted using RIPA buffer (Cat# R0278, Sigma). Nuclear or cytoplasmic fractionation of mouse fibroblasts was isolated using NE-PER® Nuclear and Cytoplasmic Extraction Reagents (Cat#78833, Thermo Fisher Scientific). Cell lysates were incubated with primary antibodies overnight at 4 °C. Protein G agarose beads (Cat# 15920-010, Life technologies) were added for another 2 h incubation at 4 °C. Samples were eluted with Laemmli sample buffer, loaded into SDS-PAGE gels, and transferred to a PVDF membrane. The membranes were blotted with 5% milk and incubated overnight at 4 °C with primary antibodies and followed by secondary antibodies. The immune complexes were detected by the enhanced chemoilluminescence (Cat# NEL103001, Perkin Elmer). In some cases, the blots were stripped and re-probed with additional antibodies. Quantification of band intensity was performed using the ImageJ (http://rsbweb.nih.gov/ij/).

### Sciatic nerve crush injury

While the mouse was under anesthesia with avertin (250 mg/kg, Cat #T48402, Sigma-Aldrich), a 1–2 cm incision just below the sciatic notch was made to expose the sciatic nerve. Forceps (Cat #11254-20, Fine Science Tools) were used to squeeze the nerve for 30 s; and turned 90 degrees to squeeze for another 30 s, which ensured that the whole nerve was crushed. Once the wound was clipped, Carprofen was administered over 3 days. NCS were performed immediately prior to the surgery and after the procedure to track the recovery of nerve function. The contralateral sciatic nerve was used as a sham control. The veterinarians and experimenters are always required to closely monitor the mouse's body weight after surgeries per IACUC protocol and found no significant weight loss. Mice among different experimental groups were evaluated simultaneously for NCS and tissue collections in nerve injury experiments.

### Statistical analysis

The data were represented as the mean ± SD. *P*-values were obtained from the Student’s two-tailed t-test or repeated-measures ANOVA. For NCS data, we used a linear mixed model where time and genotype were coded as fixed effects on CMAP and CV. A linear mixed model was also chosen to analyze the crush nerve experiments as we were interested in determining effects on continuous outcome variables and dealing with clustered data (thousands of nerve fibers in each animal for morphometric analysis, as well as repeated measurements as part of NCS). Direct comparisons between genotypes were accomplished using a two-tailed t-test. We compared continuous variables between genotype/surgery status groups for morphometric analysis using another linear mixed model with genotype and surgical status as fixed effects. All statistical analysis was performed using SPSS version 27. *P*-value < 0.05 was used to determine significance.

## Supplementary Information

Supplementary Information.

## Data Availability

All data generated or analyzed in this study are provided in the Supplementary Information/Source data file. Additional details on protocols used in this study are available from the corresponding author on request. ORS Dragonfly myelin segmentation model is also available upon request.
