# Supplementary material for: C698R mutation in Lrsam1 gene impairs nerve regeneration in a CMT2P mouse model
Source: Sci Rep. 2022 Jul 16;12:12160. doi: 10.1038/s41598-022-15902-3 (PMC9288509; doi:10.1038/s41598-022-15902-3)
Supplement: Supplementary file 1 — Supplementary Information. [file 41598_2022_15902_MOESM1_ESM.pdf]

C698R Mutation in *Lrsam1* Gene Impairs Nerve Regeneration in a CMT2P Mouse Model

Daniel Moiseev[1], Zafar Wazir[1], Donghao Liu[1], Jun Li[1, 2, 3] & Bo Hu[1*]

1. Department of Neurology, Wayne State University School of Medicine, Detroit, MI;

2. Center for Molecular Medicine & Genetics, Wayne State University School of Medicine, Detroit, MI;

3. John D. Dingell VA Medical Center, Detroit, MI

*** Correspondence:** Bo Hu, M.D., Ph.D.; Department of Neurology, Wayne State University School of Medicine, 421 E. Canfield St, 2128 Elliman Building, Detroit MI, 48201. Tel: 313-577-1586; Fax: 313-745-4216; Email: bhu@med.wayne.edu

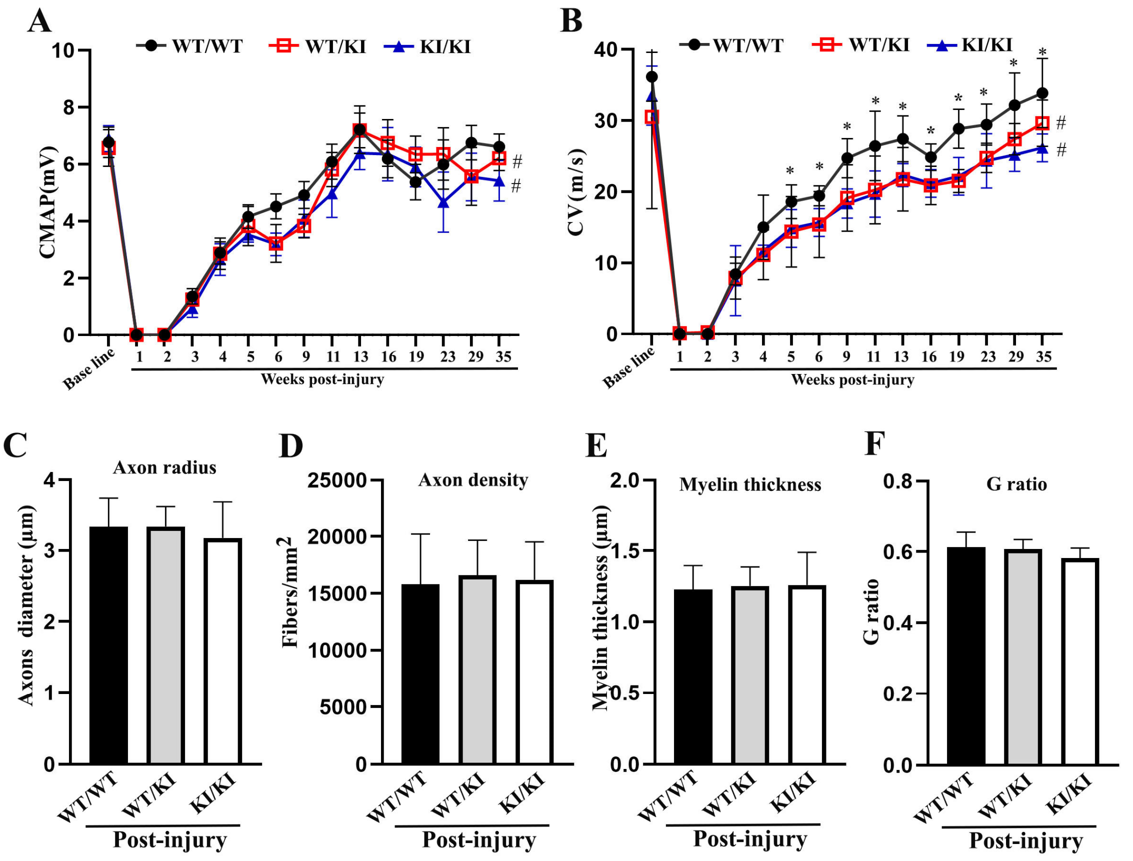

Supplementary Figure 1. Electrophysiological and morphological analysis in sciatic nerves of *Lrsam1*$^{+/+}$, *Lrsam1*$^{+/C698R}$ and *Lrsam1* $^{C698R/C698R}$ mice after nerve crush injury. (A) NCS was carried out before the surgery, post-surgery, and every 2-5 weeks until 35 weeks. Baseline measurements showed no difference in CMAP between the genotypes (n=6-7 per genotypic group, $p>0.535$). The linear mixed model showed no significant negative effect of *Lrsam1*$^{+/C698R}$ and *Lrsam1* $^{C698R/C698R}$ genotypes on CMAP. Time overall had a positive effect on CMAP, associated with 0.168mV increase per week ($p<0.001$) in all animals. #=$p<0.001$. Interactions of time and genotype were not found to be significant. (B) CV baseline measurements showed no significant difference between the genotypes (n=6-7 per genotypic group, $p>0.731$). A linear mixed model showed a significant negative effect of *Lrsam1*$^{+/C698R}$

genotype on CV: - 3.306 m/s over the whole time excluding the baseline as compared with *Lrsam1*$^{+/+}$ ($p<0.001$). *Lrsam1*$^{C698R/C698R}$ had a negative effect on CV compared with *Lrsam1*$^{+/+}$, -3.131m/s ($p<0.001$). Time overall had a positive effect on CV, associated with 760m/s increase per week overall in all animals ($p<0.001$). Interactions of Genotype x Time were found to have a significant effect on CV and indicated a slowed rate of CV recovery in *Lrsam1*$^{+/C698R}$ and *Lrsam1* $^{C698R/C698R}$ mice during the whole time period (*Lrsam1*$^{+/+}$ increased 0.869 mV ($p<0.001$), *Lrsam1*$^{+/C698R}$ increased 0.723 mV ($p<0.001$), *Lrsam1* $^{C698R/C698R}$ increased 0.671mV per week ($p<0.001$), interaction ($p<0.001$). # = $p<0.001$. We found that CV was significantly different from 5 to 35 weeks between genotypes at each timepoint post-surgery. *= $p<0.001$. (C, D, E, F) The morphometric analysis results showed no significant difference in axon radius, axon density, myelin thickness, and G-ratio in injured *Lrsam1*$^{+/C698R}$ and *Lrsam1*$^{C698R/C698R}$ nerves compared to injured *Lrsam1*$^{+/+}$ nerves nerves (n=6 per group, 4 males and 2 females each genotype).

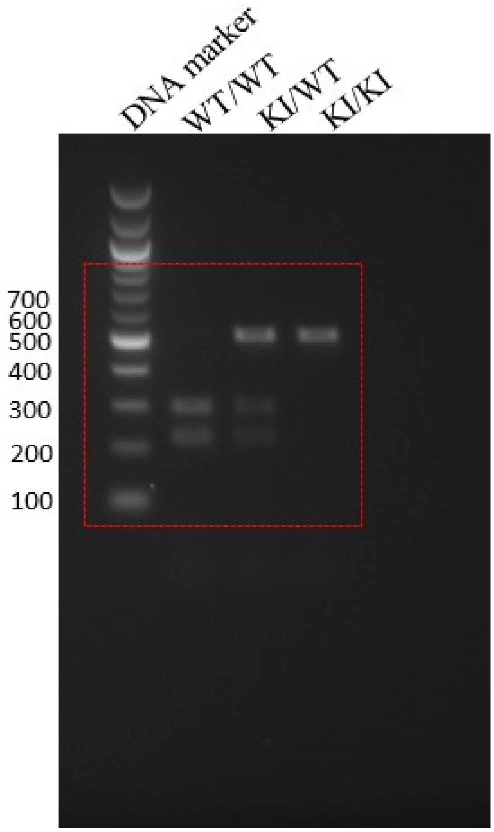

Supplementary Figure 2. Original uncropped DNA gel for Figure 1C.

The portion of the gel used for the figure is outlined with red lines.

First time co-IP experiment

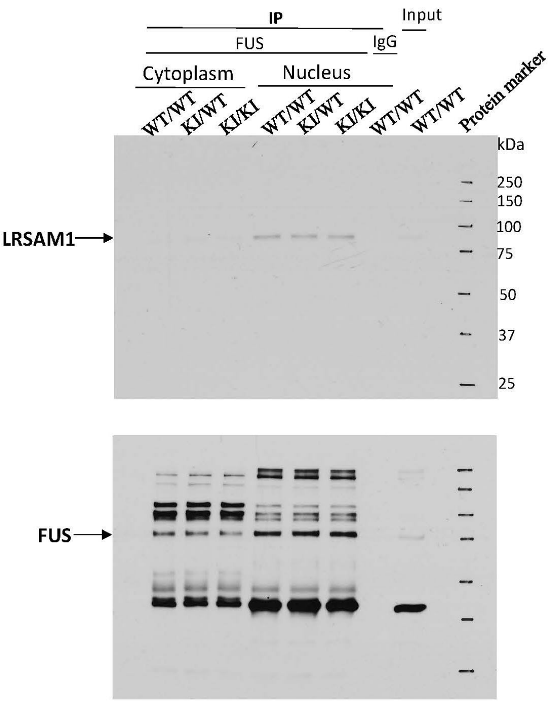

Second time co-IP experiment

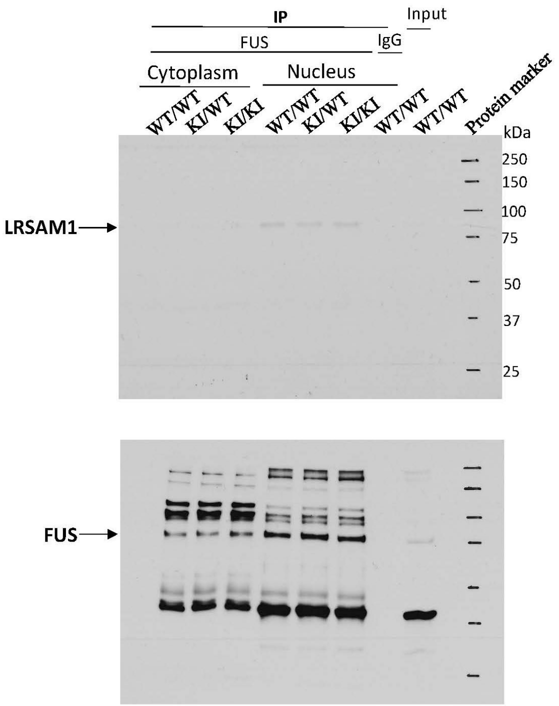

Third time co-IP experiment

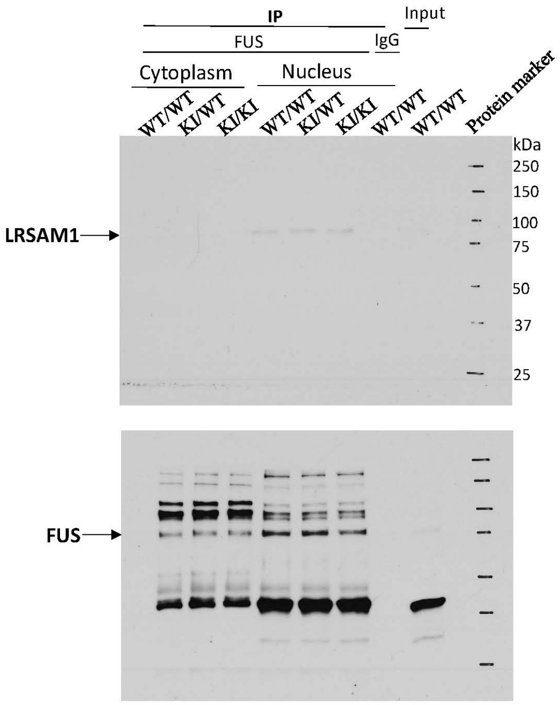

Supplementary Figure 3. Original uncropped Western blot images for Figure 5A.

First time co-IP experiment

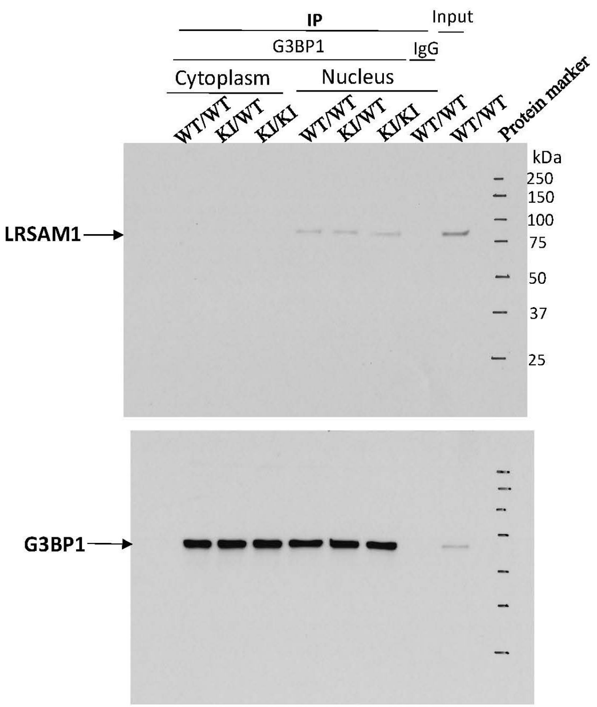

Second time co-IP experiment

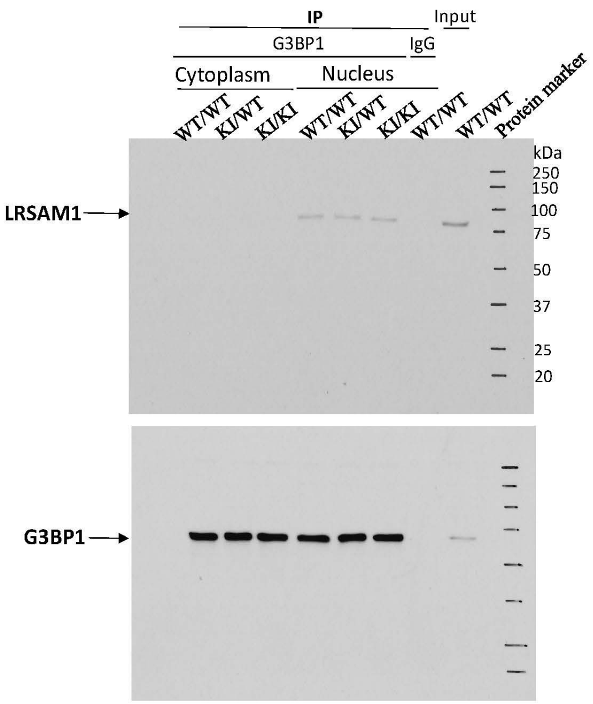

Third time co-IP experiment

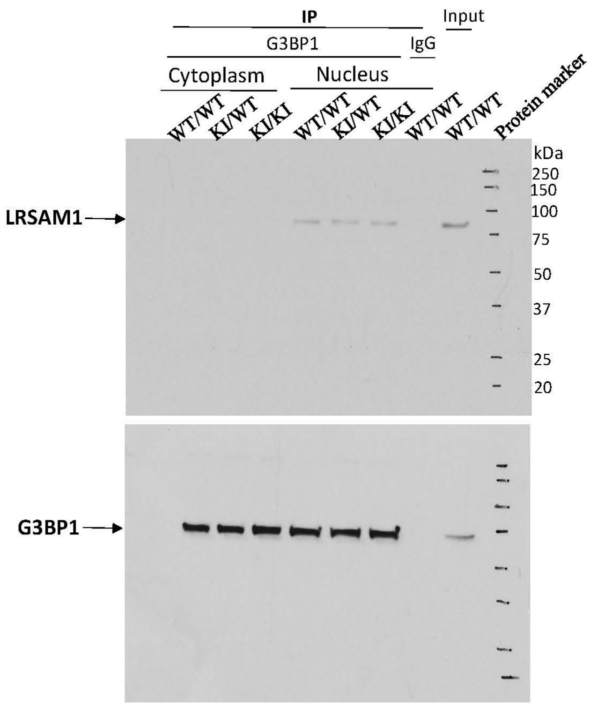

Supplementary Figure 4. Original uncropped Western blot images for Figure 5C.

First time Western blot experiment

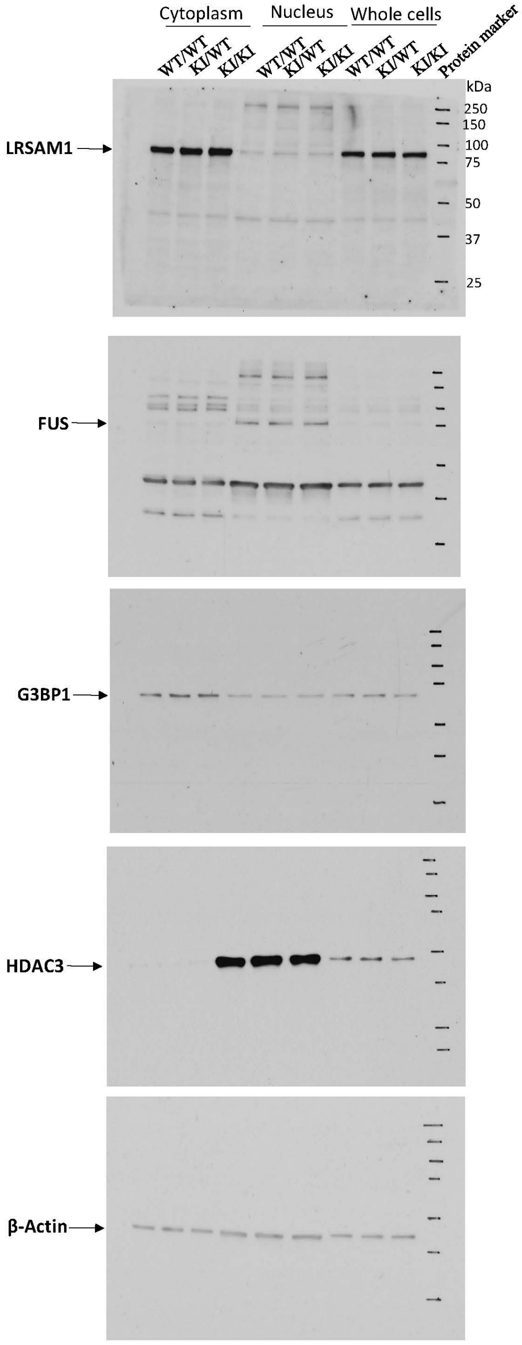

Second time Western blot experiment

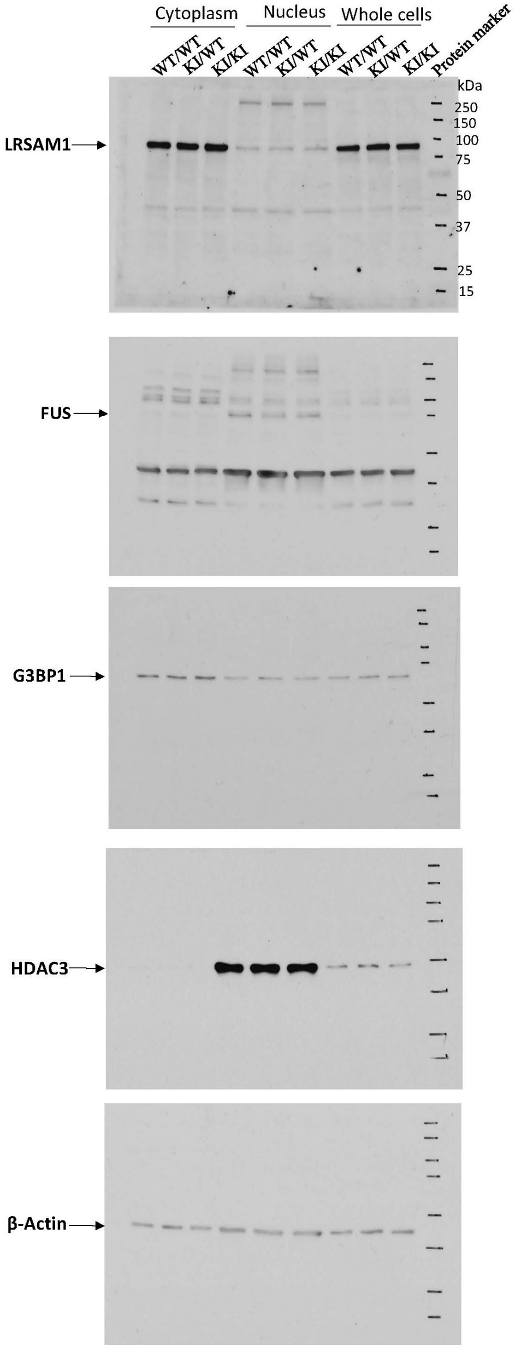

Third time Western blot experiment

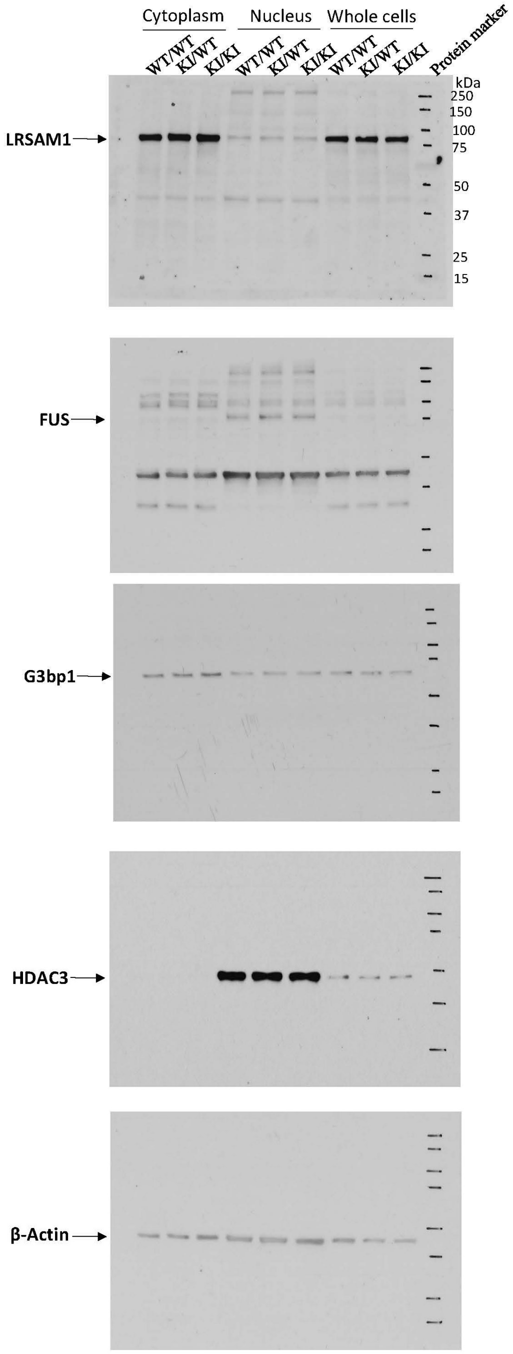

Supplementary Figure 5. Original uncropped Western blot images for Fig. 5E.
